# Structure-Guided Identification of Black Cohosh (*Actaea racemosa*) Triterpenoids with In Vitro Activity against Multiple Myeloma

**DOI:** 10.3390/molecules25040766

**Published:** 2020-02-11

**Authors:** Karin Jöhrer, Hermann Stuppner, Richard Greil, Serhat Sezai Çiçek

**Affiliations:** 1 Tyrolean Cancer Research Institute, Innrain 66, 6020 Innsbruck, Austria; karin.joehrer@tkfi.at (K.J.); r.greil@salk.at (R.G.); 2 Institute of Pharmacy, Pharmacognosy, CMBI, University of Innsbruck, Innrain 80-82, 6020 Innsbruck, Austria; hermann.stuppner@uibk.ac.at; 3 Paracelsus Medical University Salzburg, Department of Internal Medicine III, Laboratory for Immunological and Molecular Cancer Research, Müllner Hauptstraße 48, 5020 Salzburg, Austria; 4 Department of Pharmaceutical Biology, Kiel University, Gutenbergstraße 76, 24118 Kiel, Germany

**Keywords:** *Cimicifuga racemosa*, actein, sheng ma, shengmanol, cancer, tumor, natural product, cytotoxicity, apoptosis, structureactivity relationship

## Abstract

Black cohosh is a well-established medicinal plant and preparations of its rootstock are used for the treatment of mild climacteric complaints. The compounds considered responsible for the therapeutic effect are triterpene glycosides, characterized by a cycloartane scaffold and a pentose moiety. Because some of these triterpenoids were found to exhibit relevant cytotoxic effects against human breast cancer cells, we decided to investigate their activity on multiple myeloma cell lines NCI-H929, OPM-2, and U266. In a systematic approach, we initially tested three known cytotoxic compounds of three different triterpenoid types, revealing the cimigenol-type triterpenoid as the most active constituent. In a second round, seven naturally occurring cimigenol derivatives were compared with respect to their sugar moiety and their substitution pattern at position C-25, leading to 25-*O*-methylcimigenol-3-*O*-α-L-arabinopyranoside as the most potent candidate. Interestingly, not only the methyl group at position C-25 increased the cytotoxic effect but also the arabinose moiety at position C-3 had an impact on the activity. The variety of cimigenol derivatives, moreover, allowed a detailed discussion of their structure–activity relationships, not only for their effect on multiple myeloma cells but also with regard to previous studies on the cytotoxicity of black cohosh triterpenoids.

## 1. Introduction

*Actaea racemosa* (syn. *Cimicifuga racemosa*), commonly known as black cohosh, is an important medicinal plant, which was already used by the Native Americans for the treatment of rheumatism and gynecological disorders [1,2]. Presently, extracts and preparations of its rootstock are mainly applied for the alleviation of mild climacteric complaints and are an effective and safe alternative to hormone replacement therapy [3,4,5,6]. Commercial preparations consist of ethanolic or isopropanolic extracts, which are known to contain relatively high amounts of triterpenoids [7]. These triterpenoids are characterized by a 9,19-cycloartane group and considered to be the plant’s active principle [8]. Because of the hormone-like activity of black cohosh preparations, an estrogen-receptor mediated activity was initially assumed, but the alleviating effects are rather the result of GABA(A)- receptor modulation [9,10]. In the same context, black cohosh triterpenoids actein (**1**) and 23-epi-26-deoxyactein were found to exhibit beneficial effects against postmenopausal osteoporosis promoting cell growth and differentiation of osteoblast as well as osteocalcin production and mineralization [11,12,13], thus confirming the use of black cohosh for climacteric complaints [14].

Apart from the activities related to the traditional usage, several studies are dealing with the cytotoxic activity of black cohosh triterpenoids. Einbond et al., for example, investigated the impact of actein (**1**), 23-epi-26-deoxyactein and cimiracemoside A on the growth inhibition of MCF7 breast cancer cells with IC_50_ values of 14, 21 and 41 µg/mL, respectively [15]. Subsequent studies with actein (**1**) found that the inhibition of cell growth is associated with activation of specific stress response pathways and apoptosis [16] and that the compound alters the cell structure of MCF7 and MDA-MB-453 breast cancer cell lines [17]. Fang et al. investigated 23-*O*-acetylshengmanol-3-*O*-β-d-xylopyranoside (**2**), 25-*O*-acetylcimigenol-3-*O*-α-l-arabinopyranoside (**3**), 25-*O*-acetylcimigenol-3-*O*-β-d-xylopyranoside (**4**) and cimigenol-3-*O*-β-d-xylopyranoside (**6**) on MCF7 and doxorubicin-resistant-MCF7 (R-MCF7) cells, with IC_50_ values of 39 and 60 µg/mL for compound **2** and IC_50_ values between 4.0 and 5.3 µg/mL for compounds **3** and **4** [18]. The study moreover found that the p53-dependent mitochondrial signaling pathway contributes to the mechanism of apoptosis. A more recent study in a murine breast tumor-bearing model demonstrated that actein (**1**) exhibits anti-angiogenic and immunomodulatory effects and decreased tumor size and metastasis in lung and liver [19].

Because of these findings and the fact that an acetone extract of black cohosh rhizomes showed activity against different multiple myeloma cell lines, we decided to systematically investigate the effect of black cohosh triterpenoids on NCI-H929, OPM-2, and U266 myeloma cell lines. Multiple myeloma is the second most common hematologic tumor and is promoted by chronic inflammation, e.g., due to enhanced expression of TNF-alpha [20]. In the tumor microenvironment, osteoclasts are highly activated, whereas osteoblast function is blocked, and proteasome inhibitors are currently the backbone of therapy, combined with specific drugs including immunotherapeutic approaches [21]. However, this cancer is still incurable and almost all patients relapse after treatment. Therefore, novel drugs are urgently needed.

## 2. Results

### 2.1. Investigation of Different Triterpenoid Types

**Figure 1:**
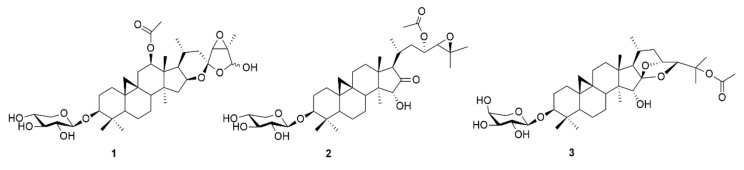
Chemical structures of actein (**1**), 23-*O*-acetylshengmanol-3-*O*-β-d-xylopyranoside (**2**) and 25-*O*-acetylcimigenol-3-*O*-α-l-arabinopyranoside (**3**).

**Figure 2:**
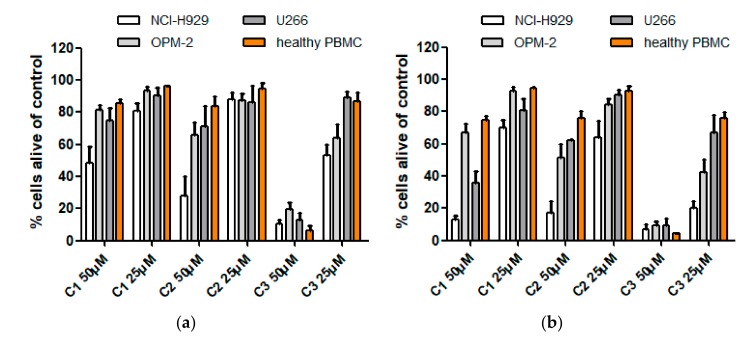
Viability of MM cell lines (NCI-H929, OPM-2, U266) and healthy peripheral blood mononuclear (PMBC) cells 24 h (**a**) and 48 h (**b**) after treatment with compounds **1**–**3**. Viability was measured by flow cytometry (AnnexinV and propidium iodide negativity) and was calculated as percentage of untreated control.

The metabolite pattern of black cohosh rhizome is unusually complex with, so far, more than 40 known different triterpenoids [7]. Because of the high number of possible candidates, in a first round three compounds representing three major triterpenoid types in black cohosh were isolated and investigated (Figure 1 and Figure 2, Table 1 and Table 2). The respective triterpenoids and triterpene types are actein (**1**, acteol-type), 23-*O*-acetylshengmanol-3-*O*-β-d-xylopyranoside (**2**, shengmanol-type) and 25-*O*-acetylcimigenol-3-*O*-α-l-arabinopyranoside (**3**, cimigenol-type).

At a concentration of 50 µM and after 24 h of incubation, actein (**1**) showed only weak to moderate cytotoxic effects, which increased after 48 h of incubation. However, at 25 µM almost no effects were observed, independently from the time of incubation. 23-*O*-acetylshengmanol-3-*O*-β-D-xylopyranoside (**2**), at a concentration of 50 µM, exhibited slightly stronger effects than actein (**1**) after 24 h of incubation and similar effects after 48 h, with the only difference that OPM-2 cells were more affected than U266 cell lines. At a concentration of 25 µM the picture was more or less the same than that of actein (**1**), with only little effects against all cell lines tested (and at both incubation times). 25-*O*-acetylcimigenol-3-*O*-α-L-arabinopyranoside (**3**) had a significantly higher impact on all three myeloma cell lines, reducing cell viability to 10% (NCI-H929), 13% (U266) and 20% (OPM-2), respectively, at a concentration of 50 µM after 24 h of incubation and below 10% for all cell lines after 48 h. In contrast to compounds **1** and **2**, the cimigenol derivative (**3**) also exhibited moderate effects at a concentration of 25 µM.

**Figure 3:**
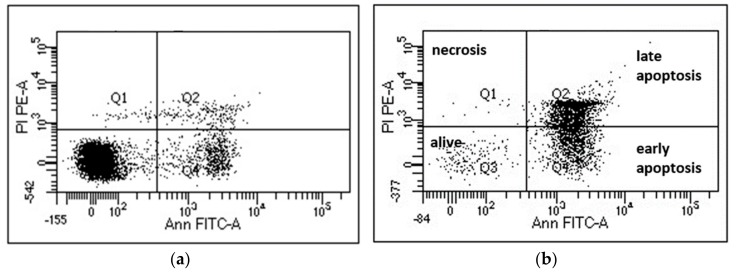
Flow cytometry analysis of NCI-H929 cells. (**a**) untreated control cells and (**b**) cells treated with 50 µM of compound **3** for 24 h. After treatment, cells were stained with AnnexinV-FITC (Ann FITC-A) and propidium iodide (PI PE-A). The percentage of cells alive (no staining) were retrieved from values in Q3.

To test whether these compounds might be able to preferentially induce cell death in cancer cells, we tested mononuclear cells from the peripheral blood of healthy donors (PBMCs) in the same setting. Promising, the average rate of cell death induction was slightly but constantly higher in the malignant myeloma cell lines compared to the healthy cells, and the extent was depending on the cell line tested. The induction of cell death is likely due to activation of apoptotic pathways since Annexin V/propidium iodide double-staining can be observed and necrosis (propidium iodide only staining) is missing (Figure 3). Induction of programmed cell death (i.e., apoptosis) is a prerequisite for a putative use as anti-cancer drug.

### 2.2. Investigation of Cimigenol-Type Triterpenoids

**Figure 4:**
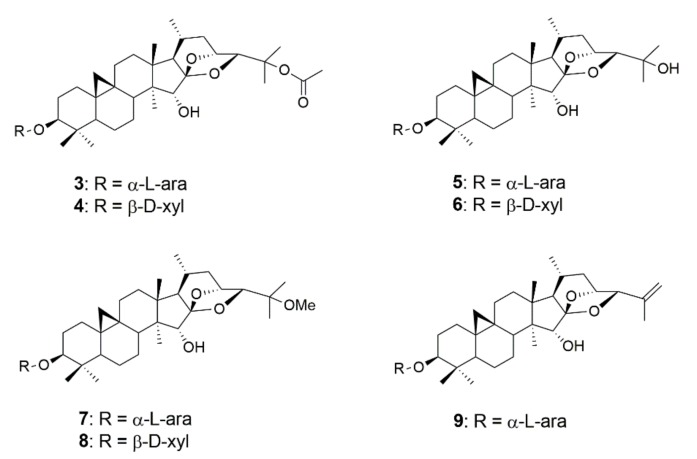
Chemical structures of 25-*O*-acetylcimigenol-3-*O*-α-l-arabinopyranoside (**3**), 25-*O*-acetylcimigenol-3-*O*-β-d-xylopyranoside (**4**), cimigenol-3-*O*-α-l-arabinopyranoside (**5**), cimigenol-3-*O*-β-d-xylopyranoside (**6**), 25-*O*-methylcimigenol-3-*O*-α-l-arabinopyranoside (**7**), 25-*O*-methyl-cimigenol-3-*O*-β-d-xylopyranoside (**8**) and 25-anhydrocimigenol-3-*O*-α-l-arabinopyranoside (**9**).

**Figure 5:**
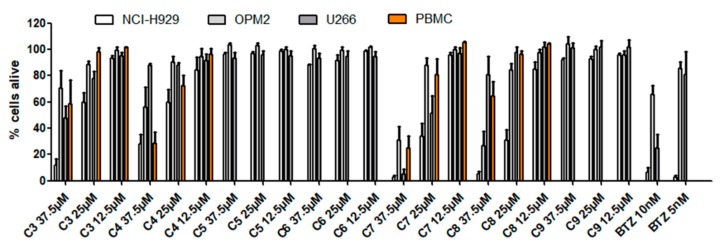
Viability of MM cell lines (NCI-H929, OPM-2, U266) and healthy peripheral blood mononuclear cells (PBMC) 24 h after treatment with compounds **3**–**9**.

In a next step, additional cimigenol-type triterpenoids isolated from *Actaea racemosa* were investigated in order to eventually identify more active constituents and to gain further insight into the structure–activity relationships of this particular compound class (Figure 4 and Figure 5, Table 3). Therefore, two positions in the molecules were of interest, the substitution pattern in position C-25 as well as the pentose moiety in position C-3. The functional groups in position C-25 comprised free hydroxy-groups, methoxy-groups, and acetoxy-groups as well as an anhydro derivative. Apart from the latter compound, all molecules were available as both α-l-arabinosides and β-d-xylosides, thus giving the opportunity to study possible effects for the two different sugar moieties. In contrast to our initial testing, incubation time was modified to 24 h and concentrations were diminished to 37.5, 25, and 12.5 µM, respectively. The effect on PMBCs was also investigated for the compounds with considerable antimyeloma activity and bortezomib, a current standard drug in myeloma treatment, was used as positive control. 

Of the seven cimigenol derivatives tested, four compounds showed considerable effects against myeloma cell lines NCI-H929, OPM-2, and U266, namely 25-*O*-acetylcimigenol-3-*O*-α-l-arabinopyranoside (**3**), 25-*O*-acetylcimigenol-3-*O*-β-d-xylopyranoside (**4**), 25-*O*-methylcimigenol-3-*O*-α-l-arabinopyranoside (**7**) and 25-*O*-methylcimigenol-3-*O*-β-d-xylopyranoside (**8**). Compounds **5** and **6**, which both consist of an underivatized cimigenol scaffold, showed no activity on any of the cell lines tested. Neither did 25-anhydrocimigenol-3-*O*-α-l-arabinopyranoside (**9**), which is characterized by the absence of a hydroxy-group in position C-25.

IC_50_ values of the four active constituents are given in Table 4 and were calculated in µg/mL to make them better comparable to previous studies [15,16,17,18]. Our selected incubation time was, however, rather short in comparison to e.g., Fang et al. who presented IC_50_ values after 48 h of incubation [18] and Einbond et al. who even used incubation times of 96 h [15,16,17]. As shown in Figure 2 as well as Table 1 and Table 2, significantly higher values of cell death were also obtained in our setting after 48 h. Nevertheless, values are still in the µM range, which might be a pitfall for in vivo application.

Compound **4** showed similar effects as its arabinose counterpart (**3**) on NCI-H929 cells, a slightly higher impact on OPM-2 cells and lower activity against the U266 cell line. Comparison of the methylated cimigenol derivatives **7** and **8** with the two abovementioned acetylated forms **3** and **4**, showed that methoxylation at position C-25 increased the activity against all cancer cell lines more than acetoxylation. For the methylated derivatives, an exchange of an arabinose with a xylose moiety did neither affect the activity against NCI-H929 nor OPM-2 cells. However, against myeloma cell line U266 the IC_50_ value of the xyloside was about twice as high (31.1 vs. 16.2 µg/mL), thus indicating that the sugar moiety contributes to the cytotoxic effect in this setting.

## 3. Discussion

*Actaea racemosa* is a well-established medicinal plant, which was investigated for its effectiveness and safety in around 20 clinical studies involving over 6000 patients [6]. Due to its frequent use in the treatment of climacteric complaints, with a daily intake of approximately 1 mg triterpenoids [7], and its thus resulting medicinal and commercial importance, black cohosh was the target of various other pharmacological and toxicological studies [14]. Most of these studies focused on extracts, fractions, or on the main compounds, such as actein (**1**) or 23-epi-26-deoxyactein. However, black cohosh not only contains a variety of different triterpenoids but also structurally very divers triterpene types (Figure 1). Acteol- and cimigenol-type triterpenoids (**1** and **3**), for example, consist of a six-ring scaffold (without counting the cyclopropane-ring), whereas shengmanol-type triterpenoids (**2**) show a four-ring scaffold and thus a more steroid-like structure. Regarding their polarity, acteol- and shengmanol-type triterpenoids show a higher grade of oxidation and therefore are usually more polar than cimigenol-type triterpenoids. This is even more pronounced, if the cimigenol scaffold underwent derivatization in position C-25 (Figure 4, compounds **3**, **4**, and **7**–**9**).

These variations in chemical structure and polarity obviously can lead to diverging activities, as observed in our earlier study on GABA(A)-receptors, where the effect of 23-*O*-acetylshengmanol-3-*O*-β-d-xylopyranoside (**2**) was exceeding that of actein (**1**) and 25-*O*-acetylcimigenol-3-*O*-α-l-arabinopyranoside (**3**) by more than the factor 5 [9]. In the present work we also observed clear differences in the activity profile of these three compounds. While compound **3** was significantly active on all three myeloma cells lines, compounds **1** and **2** only affected NCI cells remarkably (Figure 2, Table 1 and Table 2). For the other two cell lines, the effect was differential but still only moderate. Therefore, our further studies further focused on cimigenol glycosides and their derivatives.

Cimigenol-type triterpenoids represent one of the major triterpenoid classes in black cohosh and several other *Actaea* species [8,14]. In *Actaea racemosa*, cimigenol-3-*O*-α-l-arabinopyranoside (**5**) and cimigenol-3-*O*-β-d-xylopyranoside (**6**) display two of the most abundant triterpenoids [22], but also a large number of derivatives have been reported to be present in the rootstock [8]. These derivatives are characterized by acetylation, methylation, or dehydroxylation at position C-25, such as compounds **3**, **4**, and **7**–**9**, but also hydroxylation and subsequent acetylation at other positions, e.g., 1α, 7β or 12β, can be found [23]. Regarding the cytotoxicity of *Actaea* triterpenoids most interesting results, apart from actein (**1**), were obtained for cimigenol-3-*O*-β-d-xylopyranoside (**6**) and some of its acetylated derivatives [17,18]. Einbond et al. reported compound **6** to be almost as effective as actein (**1**) on cell proliferation in MDA-MB-453 (Her2 overexpressing) human breast cancer cells. In the same assay, 25-acetyl-7,8-didehydrocimigenol-3-*O*-β-d-xylopyranoside displayed the most active compound, whereupon the acetyl group at position C-25 was considered important for the growth inhibitory activity.

The importance of hydrophobic groups in position C-25 was also mentioned in a study by Fang et al. [18], in which acetylated compounds **3** and **4** were the two most active compounds against MCF7 and R-MCF7 cell lines, with IC_50_ values of 4.0 and 4.3 µg/mL (MCF7) as well as 5.3 and 4.8 µg/mL (R-MCF7), respectively. Similar activity was achieved when the acetoxy-group was replaced by chlorine; however, the fact that cimigenol-3-*O*-β-d-xylopyranoside (**6**) showed no activity in their study indicated the importance of lipophilic substitution at position C-25. 23-*O*-acetylshengmanol-3-*O*-β-d-xylopyranoside (**2**) was investigated in the same study but showed only moderate effects with about ten times higher IC_50_ values.

The results obtained in our study corroborate these findings. Similar to Einbond et al. [17] and Fang et al. [18], we found acetylated compounds **3** and **4** to be significantly more active than its hydrophilic congeners **5** and **6** and as the structurally diverse triterpenoids actein (**1**) and 23-*O*-acetylshengmanol-3-*O*-β-d-xylopyranoside (**2**). Moreover, the fact that compound **9**, which can be considered the most lipophilic compound in our study, had no effect on the cell lines tested indicates that the overall lipophilicity of the molecules is not the key criterion. It rather seems that a lipophilic group at position C-25 increases the effect in contrast to a hydrophilic group such as a hydroxyl-group, but that the absence of a functional group at this position causes a loss of activity. This can be explained with the need for electron donor groups, which are provided by methoxy- and acetoxy-groups as well as by the chlorine atom in the study of Fang et al. [18], though the latter substituent can also show electron-withdrawing properties. The fact that methylated cimigenol derivatives **7** and **8** exhibited stronger effects than acetylated compounds **3** and **4**, furthermore, suggests that the size of the functional group might as well have an impact on the activity. Having the same hydrogen accepting capacity and nearly the same polarity, methoxy and acetoxy-groups show significantly different van der Waals volumes and surface areas, which consequently affect receptor binding and specificity.

Apart from the impact of different functional groups in position C-25, our study revealed that slight differences in the sugar moiety – and thus differences at the other end of the molecule – also contribute to the antimyeloma effect of cimigenol-type triterpenoids. Of the four active components (**3**, **4**, **7** and **8**) the arabinose-bearing derivatives showed higher activity than their xylose-bearing counterparts on U266 myeloma cells, which was most evident for compounds **7** and **8**. For this cell line, the effect of the sugar moiety was even surpassing an exchange of acetyl and methyl groups in position C-25, demonstrated by the higher growth inhibitory activity of 25-*O*-acetylcimigenol-3-*O*-α-l-arabinopyranoside (**3**) compared to 25-*O*-methylcimigenol-3-*O*-β-d-xylopyranoside (**8**). L-arabinose is an epimer of d-xylose at position C-4, showing an axial hydroxy-group at this position and thus only differing by the orientation and not by the exchange of a functional group. Still, the axial orientation of the hydroxy-group leads to a slightly higher polarity and to a higher ability for eventual intermolecular hydrogen bonding.

Summarizing, in the present study the cytotoxic activity of black cohosh triterpenoids on three different myeloma cell lines was investigated. Therefore, a structure-guided approach was used to distinguish between the three most relevant triterpenoid types considering previous reports on cytotoxic effects. Subsequently, seven cimigenol-type triterpenoids were evaluated for their cytotoxic potential with respect to their substitution pattern in position C-25 as well as the sugar moiety in position C-3. The presence of several modifications, such as hydroxy-, methoxy-, acetyloxy- and anhydro-forms as well as both arabinose and xylose moieties for six out of seven derivatives allowed the systematic investigation of the necessary features for the antimyeloma activity and allowed a detailed discussion of their structure–activity relationships.

The structure-guided approach, moreover, led to the identification of 25-*O*-methylcimigenol-3-*O*-α-l-arabinopyranoside (**7**) as the most potent triterpenoid. Reports on cytotoxic activities of compound **7** are, so far, limited to a single study, in which moderate effects against human squamous cell carcinoma (HSC-2) cells and normal human gingival fibroblasts (HGF) were reported [24]. With the already known activity of black cohosh triterpenoids on human breast cancer cells and the fact that apolar cimigenol-type triterpenoids in these studies exhibited the most pronounced cytotoxic effects, further studies of 25-*O*-methylcimigenol-3-*O*-α-l-arabinopyranoside (**7**) as anti-cancer drug might be of interest. Because of the observed antimyeloma activity in our study, investigations on other hematopoietic cancer cell types, e.g., leukemia, will also be reasonable.

Apart from the identification of cytotoxic constituents in black cohosh, the present study demonstrates the need for systematic investigations in the field of phytopharmacology. In medicinal plants, different compound classes are present and even if one compound class is dominating, as e.g., in black cohosh, several structure-types may co-occur and may possess different biological activities or potencies, respectively. A successful systematic approach is shown in a previous study on copaiba, in which both the cytotoxic as well as the antimicrobial activity were attributed to certain compound classes and the necessary feature for the antifungal activity was identified [25]. This was even more important as copaiba – same as black cohosh – has a long history of medical use and therefore various reports on the biological activity of crude extracts were published. Still, only a few were dealing with pure chemically defined compounds.

This leads to another interesting aspect/challenge in phytopharmacology, where studies often focus on the investigation of extracts and/or the most abundant constituents. However, if the major components only exhibit moderate effects, it is very likely that no further experiments with minor constituents are considered necessary, especially if these constituents demand high efforts for their isolation. Thus, possible drug candidates might be overseen. Of course, when it comes to in vivo experiments, certain amounts of drug substance are required, which can hardly be obtained when the respective compound is only present in small quantities. Still, once a potential candidate is identified other ways of yielding the compound may be feasible, such as semi-synthetic approaches or isolation of the compound from closely related plant species.

## 4. Materials and Methods

### 4.1. Extraction and Isolation

#### 4.1.1. Plant Material, Reagents, and Experimental Procedures

Dried and cut plant material was obtained from Caesar and Loretz GmbH (Hilden, Germany). LC-MS grade acetonitrile, methanol and water and other (analytical grade) solvents and reagents were purchased from VWR International GmbH (Darmstadt, Germany). LC-MS grade formic acid was obtained from Sigma Aldrich Co. (St. Louis, MO, USA). Ultrasonication was accomplished with a Bandolin Sonorex ultrasonic bath (Type RK 1028 CH) and evaporation of solvents was done on a Heidolph Laborota 4000 efficient vacuum evaporator connected to a Vacuubrand PC 2001 vario pump. TLC was performed on silica gel 60 F254 plates (VWR International, Darmstadt Germany) using dichloromethane-methanol (85:15) as mobile phase and vanillin-sulfuric acid for detection. Semi-preparative HPLC was carried out on a Waters Alliance e2695 Separations Module coupled to a 2998 PDA, a 2410 RI and a WFC III fraction collector and a Phenomenex Aqua column (5 µm, 250 × 10.0 mm). Extracts, fractions, and pure compounds were analyzed by a VWR-Hitachi Chromaster Ultra RS equipped with a 6170 binary pump, 6270 autosampler, 6310 column oven, 6430 DAD and a VWR 100 evaporative light scattering detector using a Phenomenex Synergi Max-RP column (4 µm, 150 × 4.6 mm). LC-MS analysis was carried out with a Shimadzu LC-MS 8030 triple quadrupole mass spectrometer using a Phenomenex Kinetex RP-18 column (1.7 µm, 100 × 2.1 mm) and atmospheric pressure chemical ionization.

#### 4.1.2. Isolation and Identification of Investigated Compounds

Dried and ground rhizomes (700 g) were extracted three times with 5 L of acetone using ultrasonication, and the solvent was evaporated under reduced pressure to afford 42 g of a crude extract. The extract was re-dissolved in a mixture of dichloromethane-methanol (9:1) and applied to 300 g of silica gel (dry load). The sample was then subjected to silica gel column chromatography (50 × 7.5 cm) and eluted with dichloromethane-methanol (9:1 to 5:5, followed by 100% methanol) yielding six fractions (A–F). Fraction C (1.98 g) was chromatographed over a silica gel column (100 × 3.5 cm) using *n*-hexane-ethyl acetate-methanol (10:10:0.5 to 10:10:3) in a gradient manner, yielding 11 subfractions (C1–C11). Fraction C9 gave 16.38 mg of actein (1) and fraction C7 yielded 5.65 mg of 23-*O*-acetylshengmanol-3-*O*-β-d-xylopyranoside (2). Fraction B (1.17 g) was subjected to Sephadex LH-20 column chromatography (100 × 2 cm) using dichloromethane-aceton (85:15) as solvent mixture yielding 8 subfractions (B1–B8). Subfraction B4 (180 mg) was separated with semi-preparative HPLC using isocratic elution with water-acetonitrile (50:50) yielding 6.16 mg of 25-*O*-acetylcimigenol-3-*O*-α-l-arabinopyranoside (3). Compounds 4 to 9 were isolated as described in one of our previous studies [22]. Furthermore, the LC-MS method described in this study was used to confirm the identity of all isolated compounds by means of both retention times and mass spectra.

### 4.2. Cytotoxicity Assays

Induction of apoptosis was measured in myeloma cell lines NCI-H929, OPM-2, and U266 as well as in peripheral blood mononuclear cells (PBMCs) of healthy donors by flow cytometry using established protocols [26] thereby staining the cells with AnnexinV-fluorescein isothiocyanate and propidium iodide. Bortezomib (Eubio, Vienna, Austria) was used as positive control. Cell lines were purchased from DSMZ (Braunschweig, Germany) and routinely fingerprinted and tested for mycoplasma negativity. All cells (cell lines and PBMC) were grown in RPMI-1640 medium (Life Technologies, Paisley, UK) supplemented with 10% fetal calf serum (FCS; PAA, Linz, Austria), l-glutamine 100 µg/mL, and penicillin-streptomycin 100 U/mL. PBMCs from healthy donors were used after obtaining written informed consent at the University Hospital Salzburg (ethics committee approval 415-E/1287/6-2011). Cells were subjected to Ficoll separation (Ficoll PaqueTM) and incubated in RPMI-1640 Media with supplements as above. In brief, 0.5 × 10^6^ myeloma cells/mL or similar numbers of PMBCs were incubated for 24 h and 48 h with or without the tested compounds dissolved in dimethyl sulfoxide at different concentrations. At least three analyses in triplicates were performed for each cell line and a solvent control was always included. As a positive control, the proteasome inhibitor bortezomib (BTZ) was used at concentrations of 10 and 5 nM. The extent of non-apoptotic cells (AnnexinV/propidium iodide negativity) was calculated as percentage of viable cells in respect to the untreated control. Data are shown as mean percentage of viable cells and standard error of the mean (SEM) (error bars).

## 5. Conclusions

In the present study, the effect of black cohosh triterpenoids on multiple myeloma cell lines NCI-H929, OPM-2, and U266 was investigated. Following a structure-guided approach, we found apolar cimigenol-type triterpenoids to exhibit higher activity than other (more polar) triterpenoid types. Subsequent studies on cimigenol derivatives, focusing on the sugar moiety at position C-3 and the substitution pattern at position C-25, led to the discovery of 25-*O*-methylcimigenol-3-*O*-α-l-arabinopyranoside as the most potent constituent. The availability of a variety of cimigenol derivatives thereby allowed a detailed discussion of their structure-relationships and revealed an impact of the sugar moiety for one of the tested cell lines. Apart from the identification of black cohosh triterpenoids with in vitro antimyeloma activity and the structural features responsible for these effects, the present study, moreover, demonstrates the need for systematic approaches in phytopharmacology, in order to fully exploit the potential of this interesting research field.

## Figures and Tables

**Table 1:** Cytotoxicity of compounds **1**–**3** after incubation for 24 h.

		MM Cells % Alive			PBMC % Alive
cpd.	conc.	NCI-H929	OPM-2	U266	
**1**	50 µM	48.2 ± 10.9	81.1 ± 4.7	75.1 ± 6.4	85.6 ± 1.2
	25 µM	80.5 ± 5.9	93.2 ± 3.7	90.2 ± 4.1	96.4 ± 0.1
**2**	50 µM	27.9 ± 12.2	71.0 ± 11.2	56.3 ± 21.4	84.0 ± 3.0
	25 µM	87.7 ± 4.9	86.2 ± 8.6	79.4 ± 20.2	94.7 ± 1.8
**3**	50 µM	10.3 ± 2.8	19.7 ± 5.7	12.9 ± 3.5	6.1 ± 1.5
	25 µM	52.9 ± 7.1	63.8 ± 12	89.3 ± 3.2	86.5 ± 2.7

**Table 2:** Cytotoxicity of compounds **1**–**3** after incubation for 48 h.

		MM Cells % Alive			PBMC % Alive
cpd.	conc.	NCI-H929	OPM-2	U266	
**1**	50 µM	13.2 ± 2.4	67.3 ± 6.7	35.5 ± 6.7	74.9 ± 1.2
	25 µM	70.2 ± 4.3	93.1 ± 2.4	81.0 ± 5.9	94.6 ± 0.2
**2**	50 µM	17.4 ± 7.3	51.7 ± 8.9	62.1 ± 0.4	76.1 ± 2.1
	25 µM	63.9 ± 10.6	84.6 ± 1.0	90.2 ± 1.7	92.7 ± 1.7
**3**	50 µM	6.6 ± 2.2	9.6 ± 2.8	9.1 ± 3.8	4.6 ± 0.1
	25 µM	18.9 ± 3.3	43.4 ± 9.3	66.8 ± 9.5	76.2 ± 1.8

**Table 3:** Cytotoxicity of compounds **3**–**9** and bortezomib (BTZ) after incubation for 24 h.

		MM Cells % Alive			PBMC % Alive
cpd.	conc.	NCI-H929	OPM-2	U266	
**3**	37.5 µM	11.8 ± 4.3	70.8 ± 11.5	48.0 ± 7.4	58.7 ± 12.7
	25 µM	59.5 ± 7.8	88.8 ± 1.8	77.8 ± 4.6	98.3 ± 2.0
	12.5 µM	93.3 ± 1.8	99.5 ± 2.0	95.0 ± 2.4	101.7 ± 0.3
**4**	37.5 µM	27.8 ± 6.6	56.0 ± 13.0	88.0 ± 1.2	28.7 ± 5.9
	25 µM	59.5 ± 8.7	90.3 ± 3.7	87.8 ± 2.0	72.7 ± 5.4
	12.5 µM	84.3 ± 8.6	94.8 ± 5.3	91.8 ± 4.2	96.7 ± 2.9
**5**	37.5 µM	96.3 ± 1.3	103.5 ± 1.3	93.5 ± 3.5	
	25 µM	97.0 ± 1.2	103.3 ± 1.4	95.8 ± 2.8	
	12.5 µM	98.8 ± 0.9	99.8 ± 2.0	95.0 ± 3.1	
**6**	37.5 µM	88.5 ± 0.3	100.5 ± 2.4	93.5 ± 3.2	
	25 µM	91.8 ± 3.4	99.5 ± 1.8	94.5 ± 3.5	
	12.5 µM	98.5 ± 0.8	101.7 ± 0.9	94.8 ± 3.0	
**7**	37.5 µM	2.5 ± 1.3	31.3 ± 8.6	5.3 ± 2.8	25.0 ± 6.3
	25 µM	34.3 ± 8.0	88.0 ± 4.5	51.3 ± 11.8	81.0 ± 8.5
	12.5 µM	95.5 ± 1.8	99.8 ± 1.7	97.0 ± 3.5	105.3 ± 0.6
**8**	37.5 µM	5.0 ± 1.7	27.0 ± 9.4	81.0 ± 11.9	64.3 ± 8.0
	25 µM	31.3 ± 6.6	84.3 ± 4.5	97.5 ± 3.8	96.3 ± 1.7
	12.5 µM	84.8 ± 5.0	97.8 ± 2.1	102.0 ± 3.0	104.3 ± 0.3
**9**	37.5 µM	92.0 ± 1.5	104.0 ± 4.7	101.3 ± 3.3	
	25 µM	92.8 ± 1.7	100.0 ± 1.9	101.8 ± 4.3	
	12.5 µM	96.0 ±1.0	95.8 ± 2.4	101.5 ± 5.1	
**BTZ**	10 nM	6.3 ± 5.4	66.0 ± 9.4	25.0 ± 8.3	
	5 nM	3.0 ± 1.0	85.3 ± 7.6	80.7 ± 14.1	

**Table 4:** IC_50_ values of compounds **3**, **4**, **7** and **8** in µg/mL after incubation for 24 h.

cpd.	NCI-H929	OPM-2	U266	PBMC
**3**	18.1	>37.5	24.7	25.9
**4**	20.3	26.3	>37.5	20.7
**7**	14.7	21.2	16.1	19.9
**8**	14.2	20.7	31.1	26.0
